# Retraction

**DOI:** 10.3797/scipharm.ed-10-02

**Published:** 2010-03-30

On May 22^nd^ 2009 we received an article by Jignesh P. Raval, Anil J. Malaviya, Nilesh H. Patel, Hemul V. Patel and Pradip S. Patel entitled ‘Synthesis and *In Vitro* Antimycobacterial Activity of Trisubstituted *s*-Triazines’ and an external peer review was started. On June 12^th^ 2009 the managing editor accepted a revised version of the manuscript and one day later, on June 13^th^ 2009, a copy-edited version was published online [1] as ‘uncorrected proof’ in our *in press* section. Several weeks later it was brought to our intention that a very similar paper had been published in the International Journal of ChemTech Research [2]. We communicated this fact to the corresponding author, Jignesh P. Raval, and asked for clarification (especially as ambiguities in the chemical structures within each paper became evident). Unfortunately, the author failed to give reasonable explanations. Finally, the author asked for withdrawal of the article.

In conclusion, we have severe doubts about the integrity of the manuscript. Thus, the article will not be published in print. The uncorrected proof published online is retracted and the pages are watermarked ‘Retracted’.
